# Treatment of Nonclassic 11-Hydroxylase Deficiency with Ashwagandha Root

**DOI:** 10.1155/2017/1869560

**Published:** 2017-06-20

**Authors:** Daniel Powell, Taiga Inoue, Gül Bahtiyar, Gabriel Fenteany, Alan Sacerdote

**Affiliations:** Department of Medicine, New York City Health + Hospitals/Woodhull, 760 Broadway, Brooklyn, NY 11206, USA

## Abstract

An elderly woman presented with acne and male pattern alopecia, which upon diagnostic evaluation was found to be due to nonclassic 11-hydroxylase deficiency. We previously reported that Ashwagandha root ameliorates nonclassic 3-*β*-ol dehydrogenase and aldosterone synthase deficiencies. This is the first report of its use being associated with amelioration of nonclassic 11-hydroxylase deficiency, where its apparent effects appear to be dose-related.

## 1. Introduction

Congenital adrenal hyperplasia (CAH), like PCOS, is characterized by insulin resistance [1–5]. The Ayurvedic herb, Ashwagandha, (*Withania somnifera*), also known as Indian ginseng, has been used for millennia with numerous beneficial health effects having been reported; it helps fight infections, deters oil plugs from being formed on the skin, and is a natural and healthy way to treat acne scars [6, 7]. A newer usage of Ashwagandha root is as an insulin sensitizer [8]. Hyperinsulinemia may exert its redirection of adrenal steroidogenesis to androgens and away from cortisol, via its effects on the synthesis and expression of steroidogenic factor-1 (SF-1) and the nuclear transcription factor Nur77 [9]. Our group has reported a number of interventions known to decrease insulin resistance or hyperinsulinemia, which ameliorate both classic and nonclassic CAH and include metformin, thiazolidinediones, weight loss by way of lifestyle change or bariatric surgery, vitamin D replacement, and supplementation with Ashwagandha (*Withania somnifera*) [10–16]. Conversely, we have also reported that drugs known to cause insulin resistance and, in some instances, PCOS have also been shown to induce the expression of nonclassic adrenal hyperplasia [17]. Such drugs include valproate, classical and atypical antipsychotics, and antiretroviral drugs. In our previous experience with Ashwagandha, we reported that it normalized elevated serum levels of corticosterone and 17-hydroxypregnenolone and ameliorated acne and excessive scalp hair loss in a patient with both nonclassic aldosterone synthase deficiency and 3-*β*-ol dehydrogenase deficiency [14]. Here, we share our experience in treating a patient with nonclassic 11-hydroxylase deficiency with Ashwagandha.

## 2. Case Presentation

**Figure 1:**
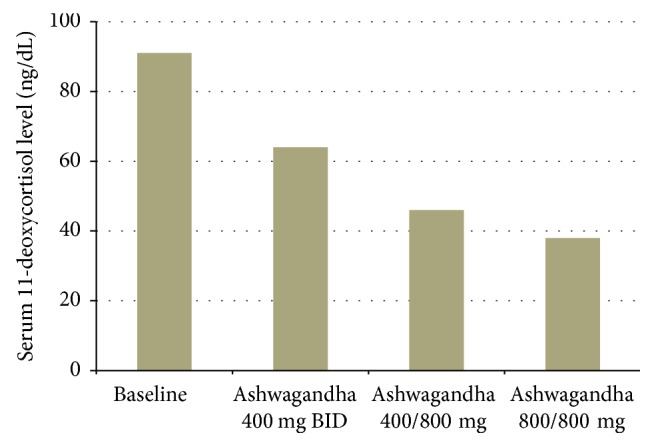
Effect of Ashwagandha root on serum 11-deoxycortisol (ng/dl).

A 78-year-old G1P1001 woman with a history of hypothyroidism, hypertension, and stage III chronic kidney disease presented with a complaint of long-standing acne and alopecia. Her growth, developmental, and reproductive history were unremarkable; her complaints began in her seventies. On 6/18/13, her unstimulated serum 11-deoxycortisol concentration was impressively elevated at 91 ng/dL (<37 ng/dL) consistent with a nonclassic 11-hydroxylase deficiency. Serum 11-deoxycortisol concentration was determined by liquid chromatography/tandem mass spectrometry. Cosyntropin (ACTH) stimulation testing was considered superfluous in this setting, as the baseline serum 11-deoxycortisol concentration was already 2.5× the upper limit of the reference range and other adrenal steroid metabolites (17-OH-progesterone, 17-OH-pregnenolone, and deoxycorticosterone) were all within normal limits and because (unlike 17-OH-progesterone) there is no quantitatively important ovarian source for 11-deoxycortisol. The patient was not treated with metformin, which is usually effective in NCAH [10, 15], because of her stage 3b chronic kidney disease. Based on our experience with other forms of nonclassic congenital adrenal hyperplasia and honoring the patient's request to try a “natural” approach, after obtaining informed consent, treatment was initiated with a standardized preparation of Ashwagandha root, with a dosage of 400 mg twice daily, which is the starting dose recommended on the product label as well as the dose that we previously reported as effective in treating other forms of NCAH [14]. On 4/9/2014, her serum 11-deoxycortisol concentration declined to 64 ng/dL. The dosage was increased to 400 mg to be taken in the morning and 800 mg in the evening. On 12/12/2014, the serum 11-deoxycortisol level further declined to 46 ng/dL. We then increased the dosage to 800 mg twice daily. On 5/3/16, the serum 11-deoxycortisol was 33 ng/dL. We noted an apparent dose/effect relationship between the Ashwagandha dosage and serum 11-deoxycortisol concentration. After a period of three years on a gradually increasing dose of Ashwagandha root, her serum 11-deoxycortisol concentration had normalized (Figure 1). Biochemical improvement has been accompanied by resolution of acne and reduction in hair loss. Since her acne was primarily facial, we chose not to photograph her, even with her eyes covered, to protect her privacy. For safety monitoring, we followed up the patient's vital signs, serum glucose, electrolytes, complete blood count, liver function tests, lipid profile, urinalysis, estimated glomerular filtration rate, and microalbumin, none of which changed appreciably.

## 3. Discussion

Ashwagandha root, often referred to as Indian ginseng, was first recorded for medical use by the Ayurveda [6, 7]. Today, it is widely grown in tropical and subtropical areas. It has been prescribed for a multiplicity of indications over several millennia and is considered by some as an adaptogen (an herb, molecule, or substance that promotes homeostasis). The first report of Ashwagandha root acting as an insulin sensitizer was by Anwer et al. [8]. As mentioned previously, we have reported amelioration of CAH through treatment with a number of insulin-sensitizing interventions including metformin, lifestyle changes, bariatric surgery, thiazolidinediones, and vitamin D repletion [10–13, 15]. Previously, we reported amelioration of two other forms of NCAH with Ashwagandha root [14]. The present report adds additional evidence to the already formidable volume of evidence that, as in PCOS, insulin resistance is not merely a bystander in CAH, but rather an integral part of its pathophysiology, thus informing an approach to its treatment. As discussed above, cosyntropin stimulation testing was unnecessary in this instance for the reasons cited in the Case Presentation; such testing is necessary, however, when there is a strong clinical suspicion of NCAH in the face of normal baseline levels of key adrenal metabolites. In such situations, cosyntropin testing may unmask those with functionally milder homozygous mutations and many heterozygotes

Withanolides are a class of compounds extracted from Ashwagandha root, and they have the ability to modulate the activity of GABAergic receptors [16]. GABAergic receptors are widely distributed in the body, including in the adrenal cortex [18]; they exert an effect on adrenal steroidogenesis [19], which could ameliorate the expression of inherited or acquired disorders of steroidogenesis. Guidelines for adult dosing of Ashwagandha have been reported [20].

Although acne in the elderly is somewhat uncommon, it is by no means unheard of [21], and when it is encountered, it is usually long-standing. Although our patient is quite elderly, there needs to be no concern about her ability to provide informed consent. She lives independently in her own home with her partner, functions independently in all activities of daily living, has no apparent cognitive impairment, and makes the 1.5-hour trip to our clinic alone by commuter train and subway. She also routinely uses the web to search for health-related information and is active on social media.

In summary, we report that Ashwagandha root may be an effective treatment for some patients with nonclassic 11-hydroxylase deficiency. Since Ashwagandha has been shown to be an insulin sensitizer, the apparent response seen here lends further support to the growing body of evidence that insulin resistance, far from being a mere side product of CAHs, is actually an integral part of their pathophysiology and a potential pathway to their treatment. As has been shown with lifestyle changes, bariatric surgery, metformin, vitamin D repletion, and thiazolidinediones, Ashwagandha use is associated with amelioration of at least some forms of CAH, without the adverse effects of glucocorticoids. The ability of withanolides to affect the activity of GABAergic receptors and the presence of the latter in the adrenal cortex raises the possibility that these compounds could ameliorate inherited and acquired defects of adrenal steroidogenesis.
